# Using AI Chatbot to Assist Students’ Behavior Management for Obesity Prevention in Middle Schools: Feasibility Study

**DOI:** 10.2196/83630

**Published:** 2026-01-07

**Authors:** Qiaoyin Tan, Yuxin Nie, Paul Son, Yizhou Qian, Amanda E Staiano, Fahui Wang, Richard R Rosenkranz, Senlin Chen

**Affiliations:** 1 School of Kinesiology, Louisiana State University, 2124 HPL Field House, Baton Rouge, LA, 70803, United States, 1 (225) 5787995, 1 (225) 5783680; 2 Pennington Biomedical Research Center, Baton Rouge, LA, United States; 3 Department of Geography & Anthropology, Louisiana State University, Baton Rouge, LA, United States; 4 Department of Kinesiology & Nutrition Sciences, University of Nevada, Las Vegas, NV, United States

**Keywords:** adolescence health, behavior intervention, large language models, school-based, mobile health

## Abstract

**Background:**

Adolescent obesity remains a pressing public health challenge, particularly among socioeconomically disadvantaged populations. Artificial intelligence (AI) holds the promise for supporting students in managing daily health behaviors, but few existing studies used AI-based interventions in naturalistic settings such as schools.

**Objective:**

This study evaluated the feasibility and preliminary impact of ProudMe Tech (Louisiana State University), an AI-assisted web app designed to help students manage 4 obesity-related behaviors: physical activity, screen time, diet, and sleep.

**Methods:**

The 8-week, 1-arm pilot intervention study recruited 172 participants from 5 middle schools in Louisiana and used the ProudMe Tech to set behavior goals, track behaviors, record reflections, and receive AI-generated feedback. Both engagement (primary focus) and behavior impacts (secondary focus) were examined.

**Results:**

Engagement metrics indicated varying levels of usage, averaging 8.9 (SD 7.6) behavior entries and 30.0 (SD 28.3) reflections per student, and receiving 33.5 (SD 29.7) AI feedback messages. Overall, participants recorded 6164 valid daily goals, of which 3934 (63.8%) were achieved. Natural language processing of the reflections and AI feedback messages revealed an overall neutral to positive sentiment. Pre- to postcomparisons showed (1) a significant reduction in screen time from 4.3 (SD 2.6) to 3.4 (SD 2.5) hours per day (21.6% decrease; *t*_164_=6.18, *P*<.001), (2) a small but significant decrease in fruit and vegetable intake from 5.7 (SD 3.8) to 5.2 (SD 3.5) servings per day (8.9% decrease; *t*_169_=2.27, *P*=.46), and (3) no significant changes in physical activity and sleep.

**Conclusions:**

These findings suggest that ProudMe Tech is a feasible AI chatbot that can engage adolescents in health behavior management, but more adaptation is needed to effectively elicit improvements in health behaviors and lower the obesity risk in middle school students.

## Introduction

The prevalence of adolescent obesity is high and demands innovative interventions. 2024 national surveillance data show that more than 20% of American youth aged 10 to 14 years have obesity, and the prevalence is even higher among racial and ethnic minorities and those from socioeconomically disadvantaged households [1]. Early adolescence marks a key developmental stage for the formation of healthy behavioral patterns, as youth have increased autonomy for physical activities, screen time, diet, and sleep—4 influential health behaviors related to obesity [2,3]. If not addressed, unhealthy behavior habits and obesity may track into adulthood [4]. Furthermore, existing behavior interventions for adolescent obesity prevention have shown limited effectiveness due to reasons such as low participation, poor personalization, and inadequate scalability [5]. Researchers have increasingly turned to novel technologies to promote behavior changes to prevent obesity. Digital tools such as mobile apps, wearable trackers, virtual or augmented reality, and gamifications have shown promising feasibility and effect in promoting physical activity, healthy diet, and sleep hygiene [6]. These technological innovations have permeated schools and homes and are naturally drawn to adolescents. Some have become important educational technologies in schools [7], but most have limited capacity to support continuous self-adjustment or personalized learning at a low cost.

Large language model-powered artificial intelligence (AI) chatbots represent an emerging and scalable technological intervention for delivering cost-effective and personalized behavioral counseling for adolescents. AI chatbots can simulate natural conversations, respond to user input in real time, and customize messages according to the user’s preferences, goals, and progress [8,9]. Early research results of adult health apps showed that compared with traditional manual guidance, AI chatbots enhanced participation and enthusiasm and improved health at a low cost [10,11]. However, little research used AI-based technology for health intervention in the younger populations (eg, for adolescent obesity prevention), especially in naturalistic settings such as schools. Rarely has prior research investigated the feasibility and impact of an AI chatbot in supporting adolescents’ management of health behaviors.

To fill the gap, this study aimed to evaluate ProudMe Tech*,* an AI chatbot that helps users (1) set specific, measurable, attainable, realistic, and timely (SMART) goals for physical activity, screen time, diet, and sleep. ProudMe Tech is part of a multicomponent intervention called ProudMe (Preventing Obesity Using Digital-assisted Movement and Eating), which was adapted from the evidence-based SWITCH-MS intervention [12,13]. The ProudMe intervention has 4 components, including (1) a 12-lesson health and physical education curriculum, (2) the simplified Smarter Lunchrooms Movement strategies, (3) professional development for teachers and staff, and (4) the ProudMe Tech. The design of ProudMe Tech is grounded in the Self-Determination Theory [14] and the Self-Regulation Theory [15] with the goal to foster users’ perceived competence, self-efficacy, and intrinsic motivation. When engaging with the ProudMe Tech, users receive informational and emotional social support from the AI as a virtual coach to improve their goal-setting, self-monitoring, and self-reflection skills to manage their health-related behaviors [16]. ProudMe Tech implementation was unsuccessful in the first round of ProudMe intervention in 2024 for unexpected reasons in reality (eg, web link blocked by the district IT, challenges with user registration, and lack of teacher buy-in) [13], but it had undergone significant refinement and beta testing. The purpose of this study was to field-test the integration of the refined ProudMe Tech into schools by evaluating its user engagement and impact on users’ health behaviors. The research team hypothesized that adolescents would actively engage with the ProudMe Tech platform and demonstrate favorable short-term changes in targeted health behaviors.

## Methods

### Setting and Participants

ProudMe Tech, as a component of the larger ProudMe intervention, was implemented in 5 public middle schools recruited within one school district from the Baton Rouge metropolitan area, Louisiana. These 5 schools primarily consisted of socioeconomically disadvantaged student populations, with a mean free and reduced-priced meal percentage of 50.4%. The ProudMe Tech intervention involved students in 6th grade, with the exception of a few 7th-grade classes. Because the ProudMe intervention was approved as an educational program, the participating teachers, at their discretion, were given authority to decide whether they would involve all students or just a selection of students. Of the 670 students taught by the participating teachers, 487 (72.7%) created a ProudMe Tech account. However, only 172 (35.3%) of those who created accounts returned parent and guardian consent forms and participated in the study. In addition, 3 students transferred to other schools during the study period, thus their partial data were removed from analysis.

ProudMe Tech was implemented in the spring semester of 2025. During the 8-week intervention period, participants accessed the ProudMe website via school-assigned laptop computers during their health and physical education classes and were expected to use the technology for at least 3 days per week. Their health and physical education teachers received standardized training at the beginning of the semester on how to use ProudMe Tech, and these teachers subsequently provided instruction for their students to create user accounts and engage with the AI chatbot. The process required limited teacher facilitation upon the initial instruction. In addition, the users were encouraged to use the technology by logging into the website beyond class time both in and out of school.

#### ProudMe Tech Website Overview 

ProudMe Tech is a web-delivered platform designed to help middle school students manage 4 health behaviors, including physical activity, screen time, diet, and sleep. Each user is expected to create a user account, set up daily goals, report their behaviors, type in a written reflection on progress toward each goal, and receive personalized feedback from the AI-powered chatbot (GPT-4). These user data subsequently generate behavior charts (line chart and progress bar chart) which offer intuitive, color‐coded visualizations of user progress relative to their goals. Users can also access daily reports which provide a calendar‐based summary of each day’s logged activities and reflections. Below provides an outline of the app interface and computer science implementation.

##### App Interface

**Figure 1.**
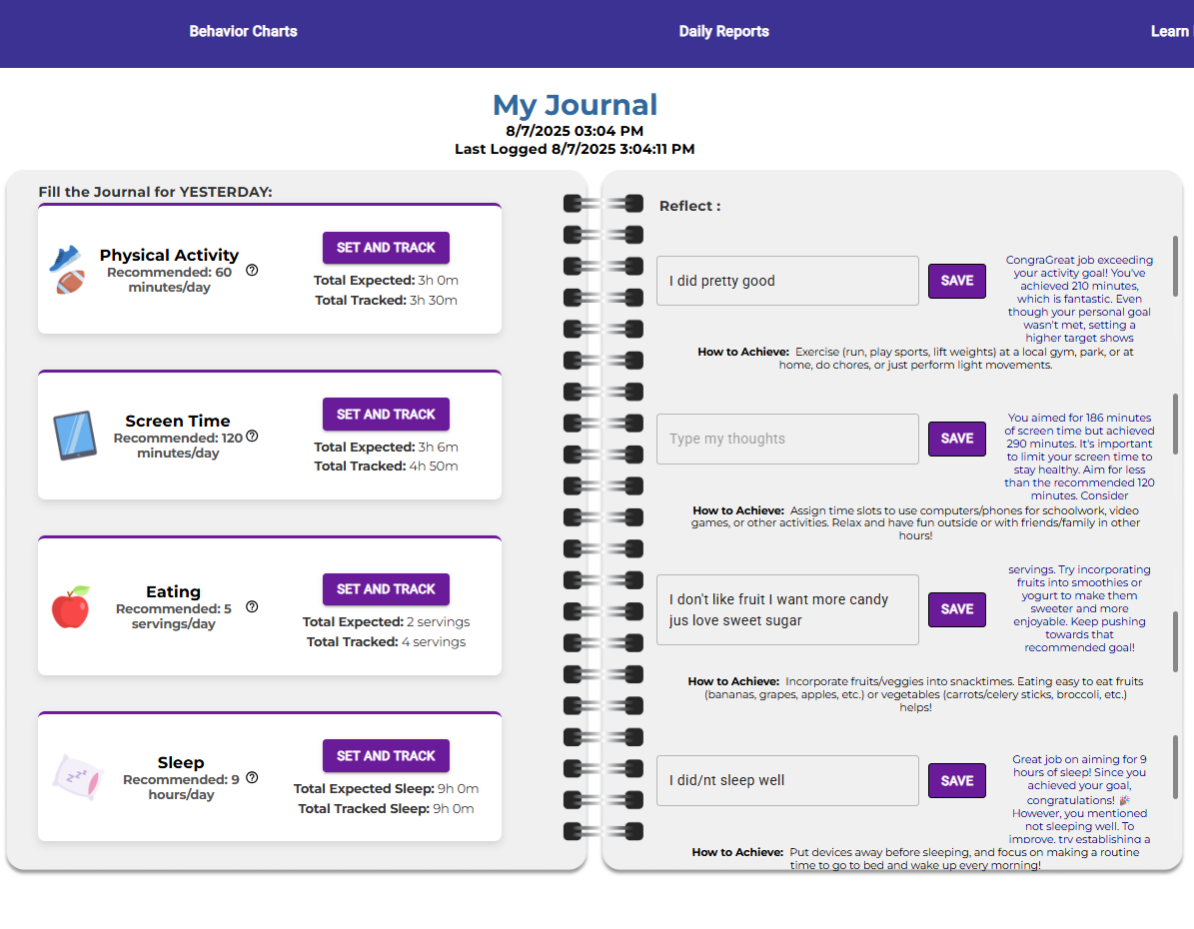
ProudMe journal.

As shown in Figure 1, on ProudMe Tech, users were instructed to create their own specific, measurable, attainable, realistic, and timely (SMART) goals and consider these principles when reflecting on goal attainment. In the first week of ProudMe Tech engagement, trained teachers taught the users of SMART goal setting and provided a demo on how to use the website app. For physical activity, users can select their activity type (eg, walking, running, basketball, and football) from a dropdown list, activity intensity (ie, strenuous, moderate, and mild), and activity volume (in hours and minutes). Screen time is categorized as academic (eg, online class and homework) and nonacademic (eg, gaming and social media) screen time. Users are prompted to select the type and amount for both goals and their actual behaviors. For diet, users tap “Add Fruit/Vegetable,” choose specific items (eg, apple and spinach) from the menu, and enter the number of servings consumed. For items not listed by default, users can manually add them. For sleep, users report their bedtime and wakeup time for both goals and actual behaviors (eg, “10:30 PM-6:45 AM”), and the system instantly computes sleep duration for users to view results prior to reflection. AI feedback was generated using a rule-based engine that matches logged behaviors to goals and delivers one of several tone-specific messages (eg, praise and suggestion).

##### Computer Science Implementation

The ProudMe Tech web app was developed using a full-stack architecture: React.js (front-end), Node.js and Express.js (back-end), and MongoDB for secure data storage. It was hosted via Render Cloud Platform and deployed using an agile development approach. The app has built-in timestamps and dropdown menus for selecting activity types, entering durations, and categorizing screen time. Back-end logs automatically track every goal, actual behavior, reflection, and AI-generated feedback message. The app has incorporated automated routines that calculate counts and timestamps directly from those logs. In addition, the platform consists of a rule-based AI chatbot module that generates and delivers feedback prompts. The research team built a simple feedback service in Node.js and Express that receives each goal or reflection a user submits. The service pulls that user’s latest data from MongoDB and then uses our OpenAI API (Application Programming Interface) key (kept in an environment variable) with the OpenAI package to call GPT4 for context-aware feedback. Key model parameters were specified to ensure reproducibility: temperature=0.9, max_tokens=75, and response_format = “json/text.” The system runs each response through OpenAI’s Moderation API to filter out any inappropriate content, then saves the full conversation plus a 0‐2 keyword score in our database. Finally, the system pushes real-time feedback to users via *Socket.io* while implementing request throttling through the bottleneck library to ensure optimal performance, security, and compliant API usage.

### Instrumentation

#### Behavior Goal, Actual Behavior, Behavior and Goal Ratio, and Behavioral Impact

Behavior goals are defined as the specific behavioral targets each student sets for themselves. Participants were prompted to enter a numeric value into the ProudMe Tech app’s “Goal” field (eg, “30min Tennis” to indicate 30 minutes of playing tennis) for each of the 4 targeted behaviors: physical activity, screen time, diet, and sleep. No default goals were provided, and students set their goals independently without preset guidance from the system. These user-set goals served as the criteria against which actual behavior was compared. Actual behavior captures what the users self-reported for each of the 4 behaviors. Similar to behavior goals, participants were prompted to self-report the type and amount of the health behaviors on the same day (eg, “45min Tennis” for 45 minutes of playing tennis). To quantify goal attainment, the research team calculated the behavior and goal ratio variable for each behavior entry recorded in the database (ratio=1 means that the goal is met; ratio >1 means that the goal is exceeded; ratio <1 means that the goal is not achieved). Finally and importantly, the research team extracted a subset of complete baseline and end point behavior entries to determine the pre-to-post behavioral impact of the ProudMe Tech.

#### User Reflection

On the app, for each behavior entry, participants were prompted to write a brief reflection on their goal attainment and how they felt about their progress. Although the raw reflections are qualitative text, the research team derived 3 quantitative measures from them: total number of reflection entries from baseline to end point, length of each entry in characters, and the timestamp of submission. These reflection data were extracted from the ProudMe Tech database without transformation. In addition, sentiment of user reflection was obtained through a sentiment analysis [17] of the users’ reflections upon their daily goals and behaviors (after viewing the feedback received from the AI chatbot). Sentiment analysis enabled us to characterize the user reflections.

#### Feedback

Feedback denotes the AI-generated feedback messages pushed to users in response to their behavior entries. The system prompts that the research team designed explicitly instructed the AI chatbot to generate tone-specific feedback according to users’ goal achievement percentages for each behavior (eg, praise for meeting goals, educational tips for partial completion, and constructive suggestions for low completion). This design ensures consistent and reproducible feedback generation across users, while also eliciting meaningful and useful feedback for the users’ behavior management. Users’ engagement data were recorded automatically in the system, which was processed in real time and then later classified by mood through sentiment analysis. Feedback was quantified using the total number and length of messages delivered to each user.

### Data Collection

Prior to data collection, the researchers informed the participants of the research purposes, procedures, benefits, and harms of the project, as well as their rights to participate in, decline, or withdraw from the study. Trained researchers handed out the consent and assent forms to the students, who were instructed to bring home the forms and seek parental consent. The same consent and assent forms were also shared virtually to parents and students by the teachers through the Google Classroom platform and email. During the 8-week school intervention, all data were automatically saved on the ProudMe Tech database instantaneously upon user entries. The participants were initially instructed to create accounts and then engage with the technology. They recorded their behavior goals, actual behaviors, reflections, and called for AI chatbot feedback. Data were extracted from the back-end database for processing and analysis.

### Data Reduction

Based on descriptive scatterplots and pre-post summaries, the authors removed extreme observations as outliers that could distort visual scales. For each domain, the 1st and 99th quantiles of the behavior and goal ratio were computed on the pooled baseline-plus-end point data. Physical activity and screen time limited to 0‐12 hours, fruits and vegetables limited to 0‐20 servings, and sleep limited to 3‐12 hours. Observations outside this range were tagged as outliers and excluded. By design, the trimming retained ≈98% of the records. Screen time goals were evaluated using reverse logic, where goals were considered achieved when actual screen use was less than or equal to the preset limit. Missing or zero goals were automatically excluded from the denominator to ensure that only meaningful goal-behavior pairs contributed to the analysis.

### Data Analyses

The authors first conducted descriptive analyses of behavior entries (mean, median, SD, maximum, minimum, frequencies, *n*, percentage, etc), including behavior goals, actual behaviors, behavior and goal ratio, users’ reflections, and feedback. The authors next analyzed the number and percentages of behavior goals met based on the goal and behavior ratio data and then visualized the goal and behavior ratio results by behavior using scatterplots on *ggplot2*. To determine the preliminary behavioral impact of the ProudMe Tech, the authors calculated the changes in behaviors from baseline to end point using absolute (Δ) and relative (Δ%) change scores:


$$\Delta =M_{\text{end point}}-M_{\text{baseline}}$$

$$\Delta \%=100\times \Delta /M_{\text{baseline}}$$

To evaluate user engagement with the ProudMe Tech platform, 2 distinct sets of user-generated text data were analyzed using natural language processing: user reflection and feedback. Each dataset underwent tailored analytic procedures to reflect its unique linguistic and functional characteristics. All the reflection and feedback data in the ProudMe Tech were analyzed using the *sentimentr* package (v2.9.0) [17] in R (R Core Team). This lexicon-based tool performs sentence-level sentiment scoring by identifying affective vocabulary, negations (eg, “not”), and intensifiers (eg, “very”) within the context of grammatical structure. Each sentence received a sentimental score ranging from −1 to +1, where higher values indicated a more positive sentimental tone. Scores were aggregated to compute a mean sentiment score per user, reflecting their overall sentimental valence [18]. This approach provides an interpretable index of the sentimental tone in users’ reflections and AI’s feedback. The lexicon-based sentiment analysis methods (such as those implemented in the *sentimentr* package) have been increasingly used and reported in health communication and psychological text analysis contexts, supporting their interpretability and reliability in short, user-generated reflections [19].

To categorize the types of feedback received from AI, the authors used a manual, rule-based keyword procedure implemented in R (*mutate(), str_detect() from stringr*) to map each message to one of 4 categories: positive reinforcement (eg, “great/awesome/congratulations/nice/excellent/cool”), constructive suggestion (eg, “try/consider/you could/might want to”), educational prompt (eg, “helps to/improves/ is important for/ is linked to/ reduces/ promotes/is key for/research shows that”), or other (no match).

To determine the preliminary impact of the ProudMe engagement on health behaviors, the authors conducted paired-samples *t* tests to compare baseline and end point outcomes across each of the 4 health behaviors.

### Ethical Considerations

This study was conducted in accordance with the Declaration of Helsinki and was approved by the Louisiana State University Institutional Review Board (IRB; protocol code IRBAM-21‐0702). Written informed consent was obtained from parents or legal guardians, and assent was obtained from all participating students. All collected data were deidentified prior to analysis and securely stored on encrypted, password-protected servers accessible only to the research team. Participants received a gift card as compensation for their participation.

## Results

Across the intervention, the authors captured 6164 goal-behavior pairings (1541 per domain), 5192 reflections, and 5804 AI chatbot feedback messages. A total of 172 participants recorded an average of 8.9 entries (mean 8.9, SD 7.6), with a median of 6 entries. About 53% (92 users) met or exceeded the median. Of the 6164 valid daily goals, 3934 (63.8%) were achieved based on their self-report. Table 1 presents the demographic characteristics of the participants, most of whom were 12 years old (129/172, 75%) in 6th grade (162/172, 94.7%) with slightly more girls (96/172, 55.8%) than boys (76/172, 44.2%).

**Table 1.** Sample distribution of age, sex, and grade level.

Characteristics	Participants (N=172)
Age (years), n (%)	
11	22 (12.8)
12	129 (75.0)
13	18 (10.5)
14	2 (1.2)
15	1 (0.6)
Sex, n (%)	
Female	96 (55.8)
Male	76 (44.2)
Grade level, n (%)	
6th	162 (94.2)
7th	10 (5.8)

**Figure 2.**
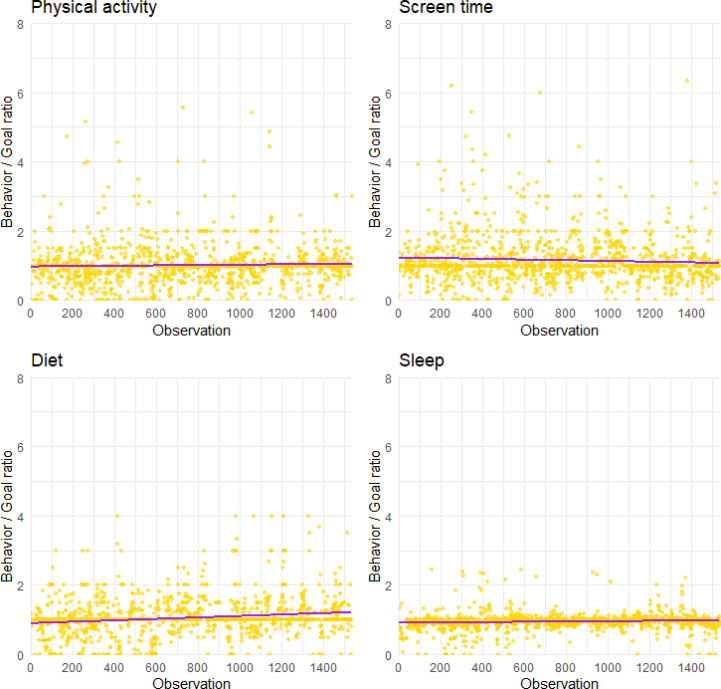
Behavior and goal ratios in 4 behaviors.

Table 2 summarizes the behavior and goal ratios across domains, showing that users on average slightly exceeded their targets. Variability was highest for screen time and physical activity and lowest for diet. Figure 2 presents the trimmed scatterplot distributions of the behavior and goal ratios for each behavior domain. Each dot represents a record of a user. The horizontal axis represents participant identifiers and the vertical axis represents the ratio of the user’s actual completion of the behavior to the set goal. The purple reference line indicates the behavior and goal ratio being 1 (goal is met but not exceeded). For physical activity, most of the observations were distributed around 1 (indicating most goals were met), some of them more than 2, indicating that some users reported abnormally high physical activity relative to their goal. For screen time (reverse), most observations were below 1, indicating that the actual screen time of users was generally lower than or close to the target value, and the goal completion was high. For diet, the observations were concentrated between 1 and 3, indicating that many users exceeded their set goals in diet (eg, eating more fruits and vegetables than their self-selected goals). For sleep, the dataset was concentrated and close to 1, indicating that the sleep behavior of most users was consistent with their goals. A correlation analysis indicated no statistically significant association between engagement frequency and self-reported goal completion rate (*r*=0.12, 95% CI –0.03, 0.26; *P*=.12).

**Table 2.** Descriptive results of the behavior and goal ratio.

Behaviors	n	Ratio, mean (SD)	Min-Max
Physical activity	1489	1.3 (1.2)	0-40
Screen time	1447	1.3 (2.0)	0-60
Diet	1471	1.1 (0.7)	0-10
Sleep	1258	1.0 (0.2)	0-2.36

**Figure 3.**
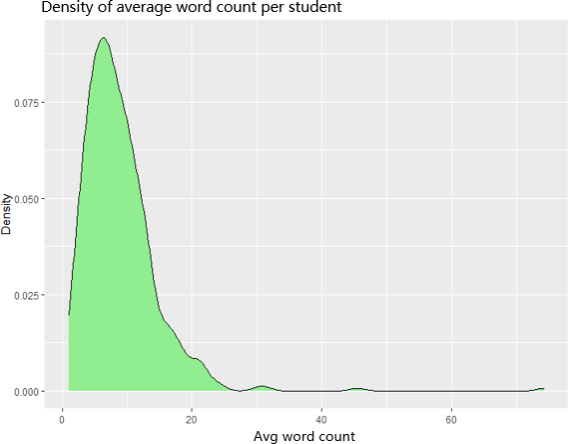
Reflection word count density.

Participants posted an average of 30 reflective notes (SD 28.3, range 1‐127; overall total of 5192), averaging roughly 10 words per entry (mean 9.9, SD 9.2). A density plot, Figure 3, was used to examine the distribution of average reflection word counts per user, here as a proxy for engagement. As shown in Figure 3, length ranged from completely blank submissions to a detailed reflection of 195 words, indicating that while most reflections were very brief, a small number of participants were considerably more elaborate and drove the wide variation in word counts (range 0‐195).

**Figure 4.**
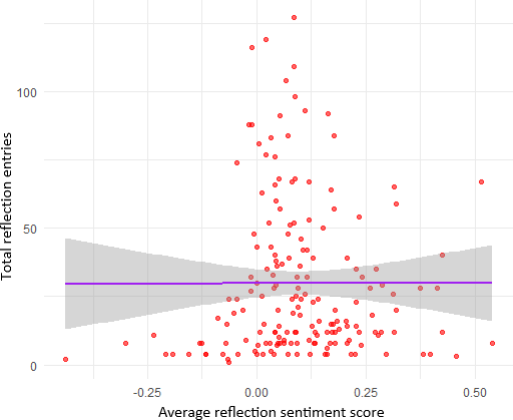
Average reflection sentiment versus total reflection.

Figure 4 plots each user’s average reflection sentiment score (*x* axis) against their total number of reflection entries (*y* axis). Each red dot represents a user’s combined data: most sentiment scores cluster around zero (neutral tone), ranging from approximately –0.25 (more negative reflections) to +0.50 (more positive reflections), while total reflections span from 0 up to over 120 entries. The purple regression line, with its wide gray 95% confidence band, lies nearly flat, indicating a statistically nonsignificant association between how positively or negatively users wrote and how often they reflected.

Table 3 presents a total of 5804 AI-generated feedback messages categorized into 4 tone types. “Positive reinforcement” dominated the chatbot output, accounting for 3493 messages (60.2%). This was followed by “Educational prompts” totaling 1595 (27.5%). Only 675 “constructive suggestions” (11.6%) and 41 “other” remarks (0.7%) were observed.

**Table 3.** Total frequency of feedback tone categories.

Feedback category	Frequency (n)	Percentage (%)
Positive reinforcement	3493	60.2
Educational prompt	1595	27.5
Constructive suggestion	675	11.6
Other	41	0.7
Total	5804	100.0

**Figure 5.**
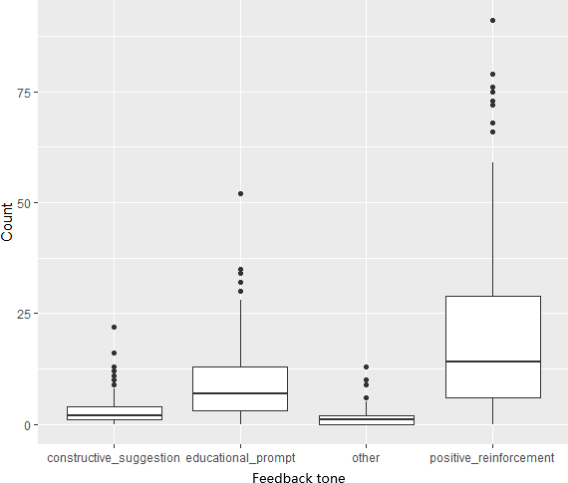
Distribution of feedback types per student.

Figure 5 depicts the distribution of feedback per user. “Positive reinforcement” was the most frequent category averaging 20.2 messages (SD 19.5) per user. “Educational prompts” was the second most frequent feedback type (mean 9.2, SD 8.8) followed by “constructive suggestions” (mean 3.9, SD 4.5,) and “Other” (mean 0.2, SD 0.5). Overall, the AI chatbot feedback skewed toward affirmation rather than instruction or corrective messaging.

**Figure 6.**
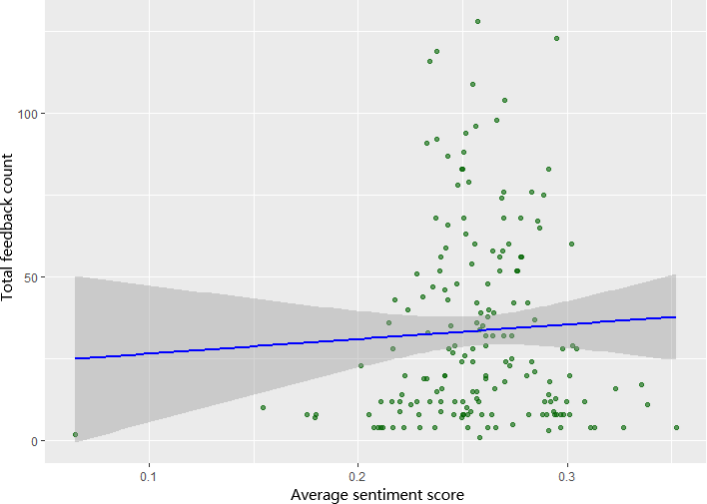
Scatterplot of average feedback sentiment versus total feedback.

Figure 6 illustrates the relationship between the average sentiment scores of AI feedback and the total number of feedback received per user. Overall, most sentiment scores were above zero, indicating a generally neutral to mildly positive tone across the feedback messages. The association between the quantity of AI-generated feedback received and the average sentiment score of that feedback was statistically nonsignificant.

In Table 4, results indicated that screen time decreased significantly from 4.3 (SD 2.6) hours at baseline to 3.4 (SD 2.5) hours at end point (*t*_164_=6.18, *P*<.001), reflecting a 21.6% reduction. Fruit and vegetable intake also declined significantly, from 5.7 (SD 3.8) to 5.2 (SD 3.5) servings (*t*_169_=2.27, *P*=.02), an 8.9% decrease. In contrast, physical activity and sleep did not significantly change (*P*=.20).

**Table 4.** Paired *t* test results of the health behaviors by time (baseline and end point).

Behavior	N	Baseline, mean (SD)	End point, mean (SD)	Δ (absolute)	Δ %	*t* test (df)	*P* value
Physical activity (hours)	169	2.5 (2.3)	2.4 (2.3)	–0.12	–4.7	1.29 (168)	.20
Screen time (hours)	165	4.3 (2.6)	3.4 (2.5)	–0.93	–21.6	6.18 (164)	<.001
Fruit and vegetables (servings)	169	5.7 (3.8)	5.2 (3.5)	–0.50	–8.9	2.27 (168)	.02
Sleep (hours)	157	7.6 (1.5)	7.7 (1.5)	+0.06	+0.8	–0.74 (156)	.46

## Discussion

### Principal Findings

The participants demonstrated sustained engagement with ProudMe Tech over the 8-week deployment, though the engagement metrics varied considerably between individuals. Results showed acceptable feasibility of the ProudMe Tech, showing an average of 8.9 behavioral entries, 30 reflections, 33.5 AI-generated chatbot feedback messages per participant, and 63.8% of the daily goals achieved. Compared to existing adolescent AI chatbots or digital health interventions (around 50% usage) [9,10], the usage and engagement metrics reported in this study were deemed acceptable. The users demonstrated a wide spectrum of engagement, with some users engaging with the platform daily, producing frequent and detailed reflections that prompted substantive feedback, while others participated sporadically, resulting in fewer and briefer interactions. Such variations in both quantity and quality of the users’ behavior entries, reflections, and AI-generated feedback appeared to be characteristic of adolescent engagement patterns as shown in similar prior digital health interventions, where individual motivation, access, and contextual factors play decisive roles [20]. It is noteworthy that engagement frequency was not associated with self-reported goal completion rate in this feasibility study. This suggests that higher engagement alone may not necessarily lead to greater goal attainment, possibly due to the brief intervention duration and variability in user motivation. Future studies with larger samples and longer follow-up periods should further explore this relationship and the underlying reasons. Notably, our data records indicated that 72.7% (487) of the students from the participating schools created accounts and engaged with the ProudMe Tech platform, but only 35.3% (172) of them were participants (with signed parental consent and assent). This limited our data analysis to a subsample, which might have constrained the feasibility and impact of the ProudMe Tech interaction. The relatively low enrollment suggests the difficulty of obtaining parental consent for studies that occur at underresourced schools. Future research should address this barrier to attain a higher participation rate to improve feasibility and success.

Beyond the user engagement metrics, the nature and quality of engagement with ProudMe Tech are meaningful to mention. The participants achieved 63.8% of their goals, eliciting positive reinforcement and educational prompts from the AI chatbot (87.7%). Meeting personal goals and receiving meaningful feedback might have fostered the users’ perceived competence, self-efficacy, and functional social support through this AI chatbot platform [14,15,21,22]. The combination of regular goal achievement and supportive feedback may have created a reinforcing loop where users have felt competent upon meeting goals and receiving affirmation, encouragement, and constructive suggestions from the chatbot. Previous studies have shown that such feedback structure, in conjunction with encouragement with actionable suggestions, can significantly increase behavior adoption and adherence in youth populations [8,9,23]. It is important to note that these feedback patterns reflect the programmed structure of the ProudMe chatbot rather than dynamic learning throughout the trial; the observed reinforcement and prompts were generated from a predefined rule-based system responding to user inputs.

Furthermore, our pre-post comparisons revealed measurable changes in 2 of the 4 health behaviors, underscoring the preliminary impact of the ProudMe Tech engagement on these behaviors. Of the 4 behaviors, screen time decreased by 0.9 hours per day (from 4.3 to 3.4 hours) and fruit and vegetable intake showed a significant decrease (8.9%), but the endpoint average score still exceeded the recommended 5 or more servings per day (ie, 5.18 servings). These changes align with prior evidence that digitally mediated self-monitoring, coupled with timely feedback, can quickly influence screen-related behaviors [24]. The unexpected decrease in fruit and vegetable intake suggests possible ceiling effect, as these participants in the present study on average reported over 5 servings per day. Intervention to promote consumption of fruits and vegetables is important, as only 18% of children between 5 and 15 years of age meet this recommendation [25]. It is also important to note that our baseline data were collected in early February and end point data in mid-May. Seasonal changes in daylight, weather, and the proximity to end-of-semester exams may have influenced students’ daily routines related to screen time and dietary intake (even sleep) behaviors independent of the intervention. These significant behavior changes in screen time suggest that these children might have been responsive to immediate environmental cues (eg, device reminders and goal prompts) and were able to make adjustments (eg, reduced recreational device use) [26].

In contrast, physical activity and sleep did not show changes in light of the ProudMe Tech engagement. These results highlight the greater difficulty of modifying these 2 behaviors as the more ingrained, effort-dependent, chronic health behaviors within a short timeframe. One possible explanation is the lack of family engagement in this feasibility study. While adolescents may express interest in being more physically active, many of the daily choices around physical activity opportunities are heavily impacted by the home environment and parental influence. Without consistent parental support, for example, providing transportation to sports and recreational activities or reminding children to stick with sleep routines, children’s capacity to make and sustain meaningful changes in these behaviors would be constrained [27]. Shifting physical activity and sleep patterns often demand overcoming environmental (eg, access to facilities and food environments) and individual barriers (eg, time constraints and motivation) [28,29] and not all of these barriers were addressed in the ProudMe Tech app.

Despite the feasibility and impact of the ProudMe Tech intervention, this study has several limitations. First, the relatively small sample size per school and low parental consent rate might have limited the generalizability of the results. However, recruiting a large sample in an intervention study like this, from underresourced schools in particular, is difficult. Our research team used various strategies to recruit and enroll a large sample. Obtaining signed parental consent without coercion from low socioeconomic families (despite incentives) is a significant reality challenge. These said, it is typical for early-stage digital health feasibility trials conducted in real-world school settings to have smaller sample sizes than fully powered randomized controlled trials. Second, our behavioral assessment as well as students’ goal setting was self-reported in nature, which is susceptible to recall inaccuracies and social desirability bias [30,31]. The young participants (~12 years old) in this study might have experienced difficulty in setting SMART goals, tracking their health behaviors, and engaging in in-depth self-reflections. Future studies should integrate objective measures (eg, accelerometers and Fitbit trackers) to more accurately capture health behaviors in this age group [30,32]. Nevertheless, the ultimate purpose of engaging these adolescent users on the ProudMe Tech platform was to educate them about behavior management and provide them with a technology-assisted tool to practice these behavior management skills. Third, the study lacked a control group and was limited to an 8-week implementation period, restricting our ability to draw strong causal inferences about the long-term effectiveness of the intervention [23]. Although having a control group with a longer intervention period to compare the behavior impact of the ProudMe Tech would be more preferable, this feasibility study was designed to primarily evaluate engagement, usability, and short-term behavioral change trends rather than efficacy. Future research with emphasis on efficacy or effectiveness of the intervention should adopt the randomized controlled trial designs [23]. Fourth, our AI chatbots primarily delivered affirmation and educational prompts and offered relatively few constructive suggestions. Although affirmation and informational prompts can increase motivation and raise awareness, the limited presence of constructive suggestions may have reduced opportunities for adolescents to receive behavior-corrective feedback or actionable strategies [18]. Therefore, refining the chatbot algorithm to balance educational information with more frequent constructive suggestions in the future could provide adolescents with not only motivational support but also practical guidance to strengthen self-regulation and long-term health outcomes [23]. Finally, the ProudMe Tech should use strategies to educate and engage parents, because parental support plays a critical role in shaping adolescents’ dietary and physical activity behaviors and reinforcing behavior changes outside of school.

In summary, our findings suggest that our AI-assisted ProudMe Tech website app can be feasibly integrated into the middle school curriculum to engage adolescents in daily behavior management and elicit meaningful behavior shifts for screen time behaviors, but further adaptations are needed to improve physical activity and sleep behaviors. ProudMe Tech provides an example for low-cost, school-based, AI-assisted health behavior interventions.

### Conclusions

These findings suggest that ProudMe Tech is a feasible AI chatbot that can engage adolescents in health behavior management, but more adaptation is needed to effectively elicit improvements in health behaviors and lower the obesity risk in middle school students.

## Abbreviations

AI: artificial intelligence
API: Application Programming Interface
SMART: specific, measurable, attainable, realistic, and timely
